# Vertebrate Ssu72 Regulates and Coordinates 3′-End Formation of RNAs Transcribed by RNA Polymerase II

**DOI:** 10.1371/journal.pone.0106040

**Published:** 2014-08-28

**Authors:** Shotaro Wani, Masamichi Yuda, Yosuke Fujiwara, Masaya Yamamoto, Fumio Harada, Yoshiaki Ohkuma, Yutaka Hirose

**Affiliations:** 1 Laboratory of Gene Regulation, Graduate School of Medicine and Pharmaceutical Sciences, University of Toyama, Sugitani, Toyama, Japan; 2 Department of Molecular and Cellular Biology, Cancer Research Institute, Kanazawa University, Kakuma-machi, Kanazawa, Japan; Keio University, Japan

## Abstract

In eukaryotes, the carboxy-terminal domain (CTD) of the largest subunit of RNA polymerase II (Pol II) is composed of tandem repeats of the heptapeptide YSPTSPS, which is subjected to reversible phosphorylation at Ser2, Ser5, and Ser7 during the transcription cycle. Dynamic changes in CTD phosphorylation patterns, established by the activities of multiple kinases and phosphatases, are responsible for stage-specific recruitment of various factors involved in RNA processing, histone modification, and transcription elongation/termination. Yeast Ssu72, a CTD phosphatase specific for Ser5 and Ser7, functions in 3′-end processing of pre-mRNAs and in transcription termination of small non-coding RNAs such as snoRNAs and snRNAs. Vertebrate Ssu72 exhibits Ser5- and Ser7-specific CTD phosphatase activity *in vitro*, but its roles in gene expression and CTD dephosphorylation *in vivo* remain to be elucidated. To investigate the functions of vertebrate Ssu72 in gene expression, we established chicken DT40 B-cell lines in which Ssu72 expression was conditionally inactivated. Ssu72 depletion in DT40 cells caused defects in 3′-end formation of U2 and U4 snRNAs and *GAPDH* mRNA. Surprisingly, however, Ssu72 inactivation increased the efficiency of 3′-end formation of non-polyadenylated replication-dependent histone mRNA. Chromatin immunoprecipitation analyses revealed that Ssu72 depletion caused a significant increase in both Ser5 and Ser7 phosphorylation of the Pol II CTD on all genes in which 3′-end formation was affected. These results suggest that vertebrate Ssu72 plays positive roles in 3′-end formation of snRNAs and polyadenylated mRNAs, but negative roles in 3′-end formation of histone mRNAs, through dephosphorylation of both Ser5 and Ser7 of the CTD.

## Introduction

In eukaryotes, all protein-coding genes and many non-coding RNA genes are transcribed by RNA polymerase II (Pol II), which consists of 12 subunits. The largest subunit of Pol II possesses the catalytic activity and also contains a unique C-terminal domain (CTD) composed of multiple repeats of the evolutionarily conserved heptapeptide sequence Tyr1-Ser2-Pro3-Thr4-Ser5-Pro6-Ser7 (YSPTSPS) [1]. The repeat number varies between species, ranging from 26 in yeast to 52 in vertebrates [1]. The CTD, which is essential for cell viability, is subjected to reversible phosphorylation during the transcription cycle, predominantly at Ser2, Ser5, and Ser7 of the repeats [2], [3]. Multiple kinases and phosphatases act on the CTD in a transcription stage-specific manner, thereby generating different CTD phosphorylation patterns along transcribed genes [2], [3]. Various nuclear factors involved in RNA processing, histone modification, and transcription elongation/termination can bind the CTD in a phosphorylation pattern-specific manner, providing a basis for coordination between transcription and other processes related to gene expression, such as histone modification and RNA processing [4]–[6].

Prior to transcription initiation, the pre-initiation complex preferentially recruits Pol II enzymes with a hypophosphorylated CTD [7]. Upon initiation, Ser5 is phosphorylated by CDK7, a subunit of the general transcription factor TFIIH [4], [6]. Phosphorylated Ser5 (Ser5P) promotes the recruitment of the capping enzyme and histone methyltransferase Set1 to the early transcription complex [4], [6]. During the transition from initiation to early elongation, Ser2 is phosphorylated by P-TEFb (CDK9/Cyclin T) [8]. As transcription proceeds from 5′ to 3′ direction, Ser2P levels are gradually increased through the actions of P-TEFb [8] and CDK12/13 [9]; concurrently, Ser5P levels decline [2], [5], [10]. Ser2P promotes the recruitment of a histone methyltransferase, 3′-end processing factors, and transcription termination factors to the elongating Pol II [2], [3], [6], [10]. TFIIH also phosphorylates Ser7 residues near promoters [11]–[13]. Ser7P participates in snRNA transcription and 3′-end processing by specifically recruiting Integrator complex and the putative CTD phosphatase RPAP2 [14], [15]. Furthermore, a recent study suggested that Thr4P is involved in 3′-end processing of replication-dependent histone mRNAs [16]. Thus, the dynamically phosphorylated CTD temporally couples transcription with other nuclear processes by serving as a scaffold for recruitment of various proteins involved in transcription, chromatin modification, and RNA processing [2], [3], [10]. Therefore, regulation of CTD phosphorylation patterns during the transcription cycle by CTD kinases and phosphatases is crucial for proper gene expression.

Ssu72 is a well-studied CTD phosphatase in yeast. The *Ssu72* (suppressor of *Sua7* 2) gene was originally identified in budding yeast as an essential gene that genetically and physically interacts with the general transcription factor TFIIB (*Sua7*) and affects the precision of transcription start site selection [17]. Subsequently, Ssu72 was shown to be a subunit of cleavage and polyadenylation factor (CPF) holo-complex that is involved in 3′-end processing of some pre-mRNAs and in transcription termination of small non-coding RNAs such as snoRNAs, snRNAs, and cryptic unstable transcripts (CUTs) [18]–[22]. Although yeast Ssu72 was initially shown to be a Ser5P-specific CTD phosphatase [23], recent studies have demonstrated that it also exhibits Ser7P phosphatase activity *in vitro*. Consistent with this, loss of Ssu72 *in vivo* results in an increase in the phosphorylation level of both Ser5 and Ser7, both at snoRNA genes and in the 3′ regions of mRNA genes [24], [25].

The mammalian ortholog of yeast Ssu72 was originally identified as a binding partner of the tumor suppressor RB [26]. Although mammalian Ssu72 is very similar at the sequence level to yeast Ssu72 and can also associate with TFIIB, it is unable to rescue a lethal *ssu72* mutation in yeast, and its suppression does not affect cell proliferation or viability of mammalian cultured cells [26]. Thus, mammalian Ssu72 may share a subset of the functions of the yeast protein but also exert specific functions in mammalian cells. Although recent studies demonstrated that human Ssu72, like its yeast counterpart, exhibits Ser5P and Ser7P-specific CTD phosphatase activity *in vitro* [27], [28], its *in vivo* roles in CTD dephosphorylation and gene expression remain to be elucidated.

To investigate the functions of vertebrate Ssu72 at the cellular level, we developed chicken DT40 B-cell lines [29] in which Ssu72 expression is conditionally inactivated. Ssu72 depletion caused defects in 3′-end formation of U2 and U4 snRNAs and *GAPDH* mRNA. Unexpectedly, however, Ssu72 inactivation increased the efficiency of 3′-end formation of non-polyadenylated replication-dependent histone mRNA. Furthermore, Ssu72 depletion caused a significant increase in both Ser5 and Ser7 phosphorylation on all genes in which 3′-end processing was affected. These results suggest that vertebrate Ssu72 plays positive roles in 3′-end formation of snRNAs and polyadenylated mRNAs, but a negative role in 3′-end formation of histone mRNAs, by dephosphorylating both Ser5P and Ser7P of the RNA Pol II CTD.

## Results

### Establishment of conditional Ssu72-knockout DT40 cell lines

**Figure 1:**
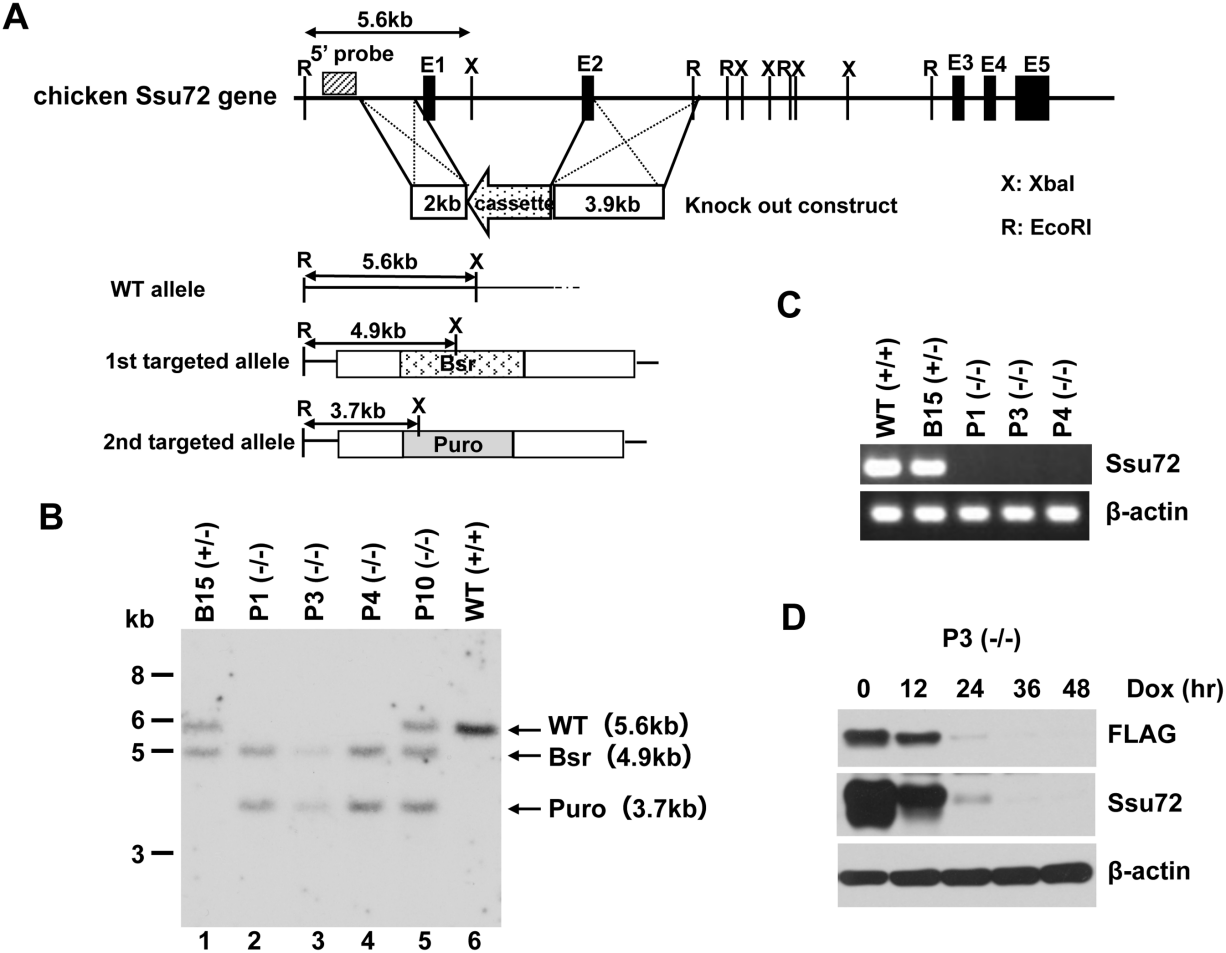
Establishment of conditional Ssu72-knockout DT40 cell lines. (A) Schematic representations of the chicken Ssu72 genomic fragment, knockout constructs, and configuration of the targeted alleles. Exons are shown as black boxes (E1–5), and the location of the 5′ probe used for Southern blotting is shown as a grey box. The double-headed arrows above the genes indicate length (in nucleotides). The *XbaI* and *EcoRI* restriction sites are indicated by vertical lines labeled X and R, respectively. (B) Southern blot analysis of wild-type (WT), heterozygous mutant (B15), homozygous mutant (P1, P2, P3), and unanticipated rearranged mutant (P10) clones. Genomic DNA obtained from each clone was digested with *Xba*I and *Eco*RI, and then hybridized with the 5′ probe shown in panel A. (C) RT-PCR analysis of the wild-type and mutant clones using primer pairs specific for the indicated gene. (D) Immunoblotting analysis of DT40 P3 (−/−) whole-cell extracts treated with Dox for the indicated times, using the indicated antibodies. Western blotting of β-actin was used as to confirm equal protein loading.

To investigate the Ssu72 functions in vertebrate cells by gene targeting, we utilized the chicken B-cell line DT40, which exhibits exceptionally high homologous recombination rates and has been widely used as a model cell line for genetic studies [29]. Because our initial attempts to establish homozygous knockouts failed repeatedly, we concluded that Ssu72 is essential for cell viability. Therefore, we generated DT40 cell lines in which Ssu72 expression was conditionally inactivated using the Tet-off regulatory system. To prepare the targeting constructs, we PCR-amplified two genomic DNA fragments, a 2 kb region upstream of the first exon of the Ssu72 gene (ENSGALG00000001489) and a 3.9 kb region downstream of the second exon, and then cloned these fragments into the flanking regions of drug-resistance cassettes (Fig. 1A). These knockout constructs were sequentially introduced into a parental DT40 cell line that stably expresses the Tet repressor fused to the herpes simplex virus VP16 activation domain, as well as a chicken Ssu72 transgene under control of the tetracycline-repressive promoter (*Ssu72* ^+/+^/FLAG-*Ssu72*). Hereafter, we refer to this parental strain as WT (+/+).

After the second round of gene targeting, we established three independent cell lines whose two wild-type alleles of the *Ssu72* gene were replaced by targeting constructs, but which still expressed the Tet-repressible Ssu72 transgene (*Ssu72* ^−/−^/FLAG-*Ssu72*). In this report, we refer to these lines as P1 (−/−), P3 (−/−), and P4 (−/−). Southern blot analysis revealed that the first (lane 1) and second (lanes 2–4) wild-type alleles of the Ssu72 gene had been successfully targeted (Fig. 1B). Expression of Ssu72 was completely abolished at the RNA level in the three homozygous mutants (Fig. 1C). Protein expression from the Ssu72 transgene in one of the homozygous mutant cell lines, P3 (−/−), was tightly repressed within 2 days after doxycycline (Dox) administration (Fig. 1D), and the kinetics of Ssu72 repression following Dox addition were similar in the other two mutants, P1 (−/−) and P4 (−/−) (data not shown).

### Ssu72 is essential for DT40 cell growth

**Figure 2:**
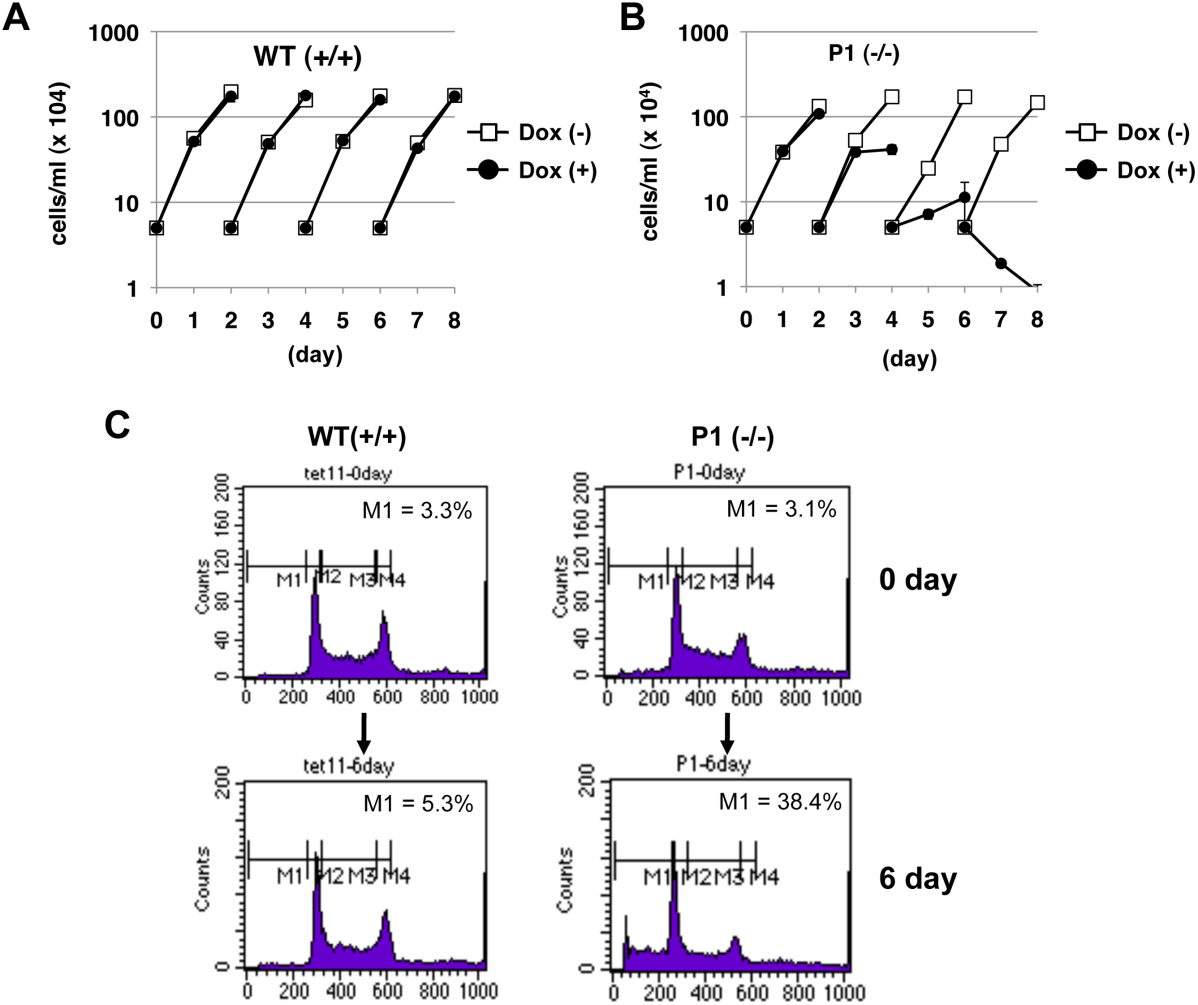
Ssu72 is essential for cell proliferation and viability in DT40 cells. (A, B) Growth curve of DT40 wild-type (WT) and conditional mutant [P1 (−/−)] DT40 cells. WT (A) or P1 (−/−) (B) cells were seeded in triplicate in 12-well plates (5×10^3^ cells/well) in media with or without doxycycline (Dox), and split every 2 days. Concentrations of live cells were determined by Giemsa staining and counted at the indicated time points. Cell density is shown on a logarithmic scale. (C) WT cells (left) or P1 (−/−) cells (right) treated with Dox for 6 days (lower) or untreated (upper) were subjected to FACS analysis after staining with propidium iodide. Gates M1, M2, M3, and M4, delimited by the vertical bars above the FACS traces, indicate the cell-cycle distributions of the sub-G1, G0/G1, S and G2 populations, respectively.

Ssu72 is highly conserved among eukaryotes and essential for yeast viability [17]. However, in mammals, siRNA-mediated knockdown of Ssu72 expression does not affect cell proliferation or viability [26]. To investigate the functional roles of Ssu72 in proliferation of vertebrate cells, we used the conditional knockout DT40 mutant cells described above to examine the effect of Ssu72 depletion on cell growth and viability. In the absence of Dox, the proliferation rate of the P1 (−/−) cell line was comparable to that of WT (+/+) (Figs. 2A and 2B, open squares). However, in the presence of Dox, the growth rate of this mutant was markedly reduced after 3 days, and the mutant cells died 6 days after Dox administration (Fig. 2B, filled squares). The other two mutants, P3 (−/−) and P4 (−/−) also exhibited similar growth patterns (data not shown). Proliferation of the mutants recovered if Dox was removed from culture media, resulting in restoration of Ssu72 protein expression (data not shown). The Dox concentration used in this study did not affect cell growth of WT (+/+), as shown in Fig. 2A.

We also examined the effects of Ssu72 depletion on cell-cycle distribution by fluorescence-activated cell sorting (FACS) analysis. Dox treatment for 6 days significantly increased the proportion of the sub-G1 population in the conditional mutants (from 3.1% to 38.4%), but not in wild-type cells (from 3.3% to 5.3%) (Fig. 2C), suggesting that Ssu72 depletion may induce apoptosis in DT40 cells. Taken together, these results suggest that Ssu72 is essential for cell proliferation and viability, at least in cultured chicken cells.

### Ssu72 depletion causes a modest increase in CTD phosphorylation

**Figure 3:**
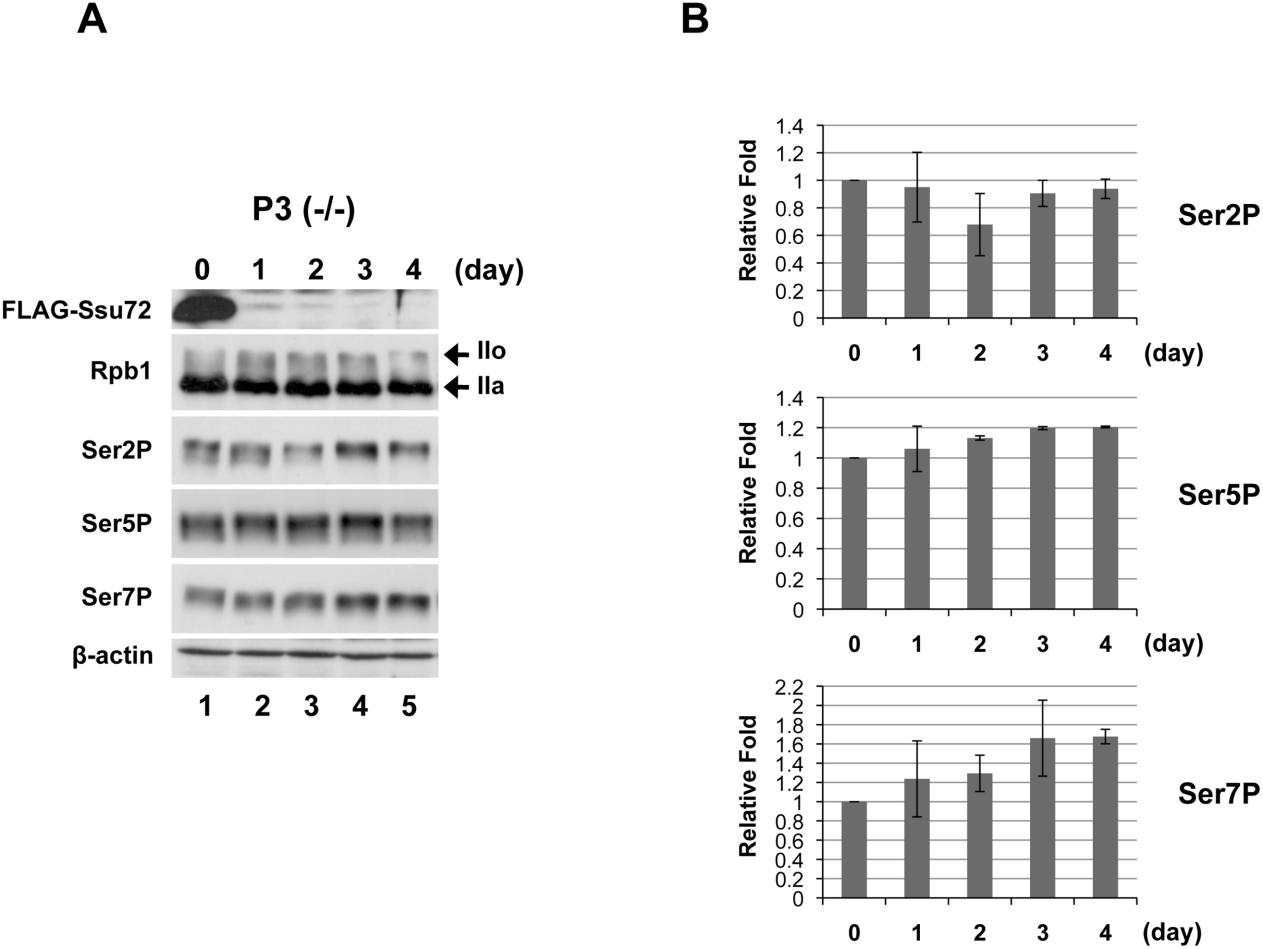
Ssu72 depletion results in a modest increase in CTD phosphorylation. (A) Whole-cell extracts from DT40 P3 (−/−) cells treated with Dox for the indicated number of days were separated by SDS-PAGE, and then analyzed by Immunoblotting with antibodies against FLAG-Ssu72 (anti-FLAG M2), the largest subunit (Rpb1) of Pol II (N20), Ser2P (3E10), Ser5P (3E8), Ser7P (4E12), and β-actin. Western blotting of β-actin was used to confirm equal protein loading. The positions corresponding to the hyper-phosphorylated form of Rpb1 (IIo) and hypo-phosphorylated form of Rpb1 (IIa) are indicated by arrows. (B) Fold change of phosphorylation levels of each serine (Ser2/5/7P) relative to the corresponding levels on day 0. The Western blot signals were quantified by using a luminescent image analyzer LAS-4000 mini (Fujifilm). The each value was normalized by total Pol II signal (Rpb1). Error bars represent standard deviations of two independent experiments.

Yeast Ssu72 exhibits CTD phosphatase activity toward Ser5P and Ser7P both *in vitro* and *in vivo* [24], [25]. Although human and fly Ssu72 also exhibit Ser5P and Ser7P phosphatase activity *in vitro* [27], [28], [30], [31], the involvement of Ssu72 in dephosphorylation of the phosphorylated CTD *in vivo* has not been examined in vertebrate cells. Therefore, we next investigated whether Ssu72 depletion affects the CTD phosphorylation level *in vivo*. Total proteins extracted from P3 (−/−) cells every 24 hours after Dox addition were subjected to Western blotting using phosphorylation-dependent monoclonal antibodies specific for Ser2P (3E10), Ser5P (3E8), and Ser7P (4E12) [32]. Total levels of the largest subunit of Pol II (Rpb1) were detected using antibodies against the N-terminal region of Rpb1 (N-20) (Figure 3A). Quantification of Western blots by using a luminescent image analyzer revealed that Ssu72 depletion caused a gradual increase of Ser5/7P levels whereas Ser2P levels were relatively constant after Dox addition (Figure 3B). Significantly, Ser7P levels at day 4 increased about 1.7-fold whereas Ser5P levels increased 1.2-fold. These results suggest that Ssu72 may contribute to Ser7P dephosphorylation more significantly than Ser5P *in vivo*. In a previous report, Ssu72 depletion in budding yeast resulted in a significant increase of the Ser5P level on total Pol II *in vivo* [23], indicating that Ssu72 may be a major Ser5P-CTD phosphatase. However, in chicken cells, Ssu72 depletion exerted only a modest effect on the phosphorylation levels of Ser5. These results suggested that in vertebrate cells, although Ssu72 may participate to some extent in dephosphorylation of Ser5P, this serine residue is primarily dephosphorylated *in vivo* by other CTD phosphatases, such as SCP1.

### Ssu72 is required for efficient 3′-end formation of snRNAs and mRNA in DT40 cells

**Figure 4:**
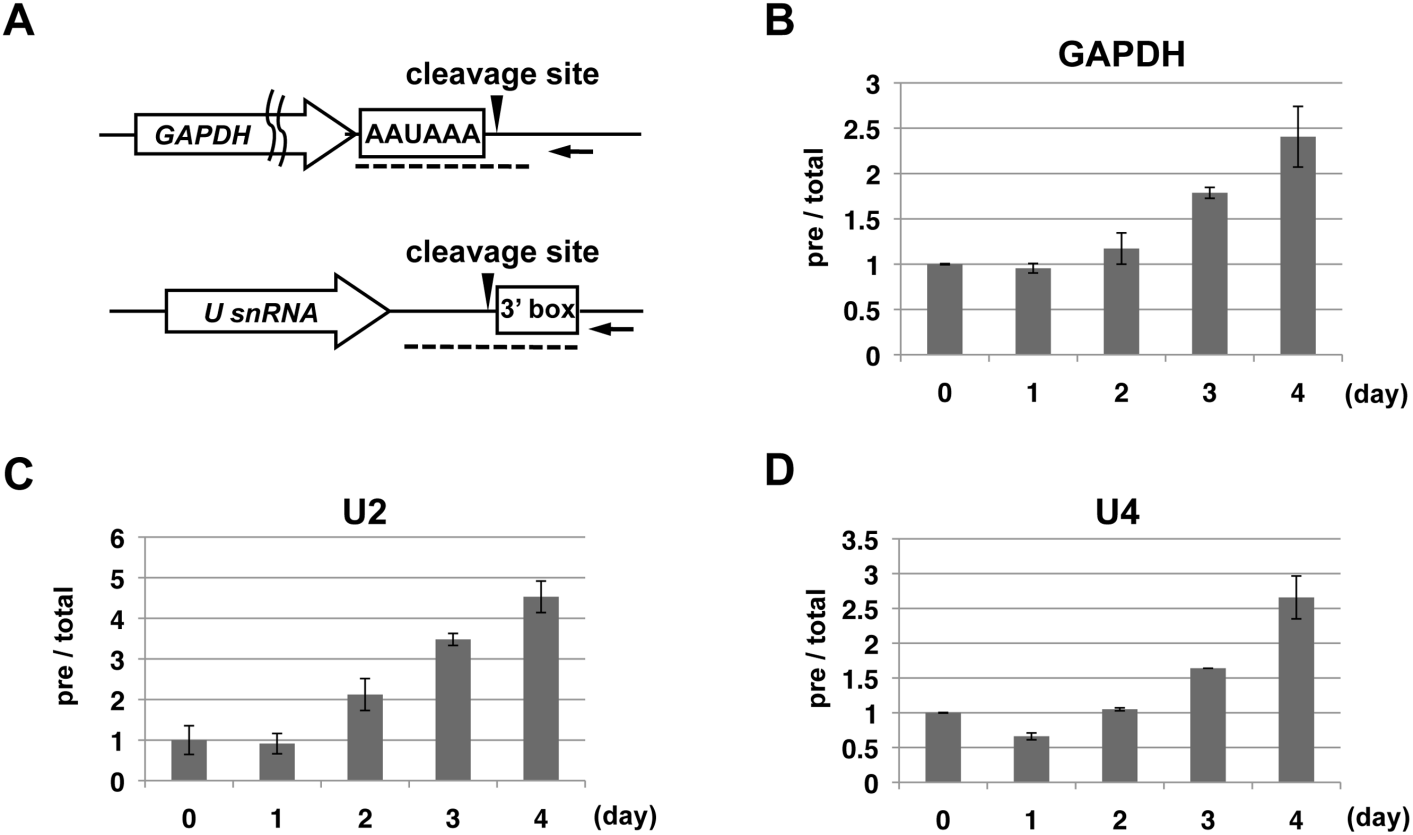
Ssu72 is required for 3′-end formation of *GAPDH* mRNA and U2 and U4 snRNAs. (A) Diagrams of the *GAPDH* (upper) and *U snRNA* genes (lower), shown as open arrows, with the cleavage sites depicted as arrowheads and RNA processing elements indicated in the open boxes. The black arrows represent the gene-specific primers used for reverse transcription of precursor transcripts. Dotted lines indicate the RT-qPCR amplicons used to quantitate the precursor transcripts. (B–D) RT-qPCR analysis of the relative expression levels of precursor (pre) and total transcripts of *GAPDH* (B), U2 snRNA (C) or U4 snRNA (D) in DT40 P3 (−/−) cells treated with Dox for the indicated number of days. Relative expression levels were normalized the corresponding levels on day 0. Error bars represent standard deviations of at least two independent experiments.

In yeast, Ssu72 participates in 3′-end formation of small non-coding RNAs transcribed by Pol II such as snoRNAs, snRNAs, and CUTs *in vivo* [18]–[21]. Yeast Ssu72 is also implicated in 3′ processing of specific mRNA both *in vitro* and *in vivo* [18]–[22]. Although a recent *in vitro* study demonstrated that mammalian Ssu72 is involved in transcription-coupled polyadenylation of model pre-mRNAs [28], it remains unclear whether vertebrate Ssu72 is involved in 3′-end formation of Pol II transcripts *in vivo*. Using the mutant DT40 cell lines, we next investigated whether Ssu72 inactivation affects 3′-end formation of two types of Pol II transcripts, poly(A)-containing mRNA and spliceosomal snRNA, whose 3′ ends are formed by distinct mechanisms (Fig. 4A) [5]. To precisely evaluate the efficiency of the 3′ processing of Pol II transcripts, we measured the expression levels of both total and unprocessed RNAs by quantitative RT-PCR analyses, and then determined the ratio of unprocessed RNA to total RNAs (i.e., precursor + mature RNAs). To detect unprocessed RNAs, we synthesized first-strand cDNAs by reverse transcription using a primer complementary to sequences downstream of the processing site in each precursor RNA (Fig. 4A, arrows), and then PCR-amplified the cDNAs using primer pairs spanning the processing sites (Fig. 4A, dashed lines). Total RNA levels were detected by amplification of coding regions from cDNAs synthesized using random hexamers.

To investigate the effects of Ssu72 depletion on 3′-end formation of Pol II genes, we first examined *GAPDH* mRNA and U2 and U4 snRNAs (Fig. 4). In metazoans, the 3′ end of poly(A)-containing mRNA is formed by endonucleolytic cleavage of pre-mRNA 20–30 nucleotides (nts) downstream of the poly(A) signal sequence, followed by polyadenylation [5]. By contrast, the primary snRNA transcript is processed by endonucleolytic cleavage just upstream of the 3′ box sequence (Fig. 4A) [14]. We isolated RNA from P3 (−/−) cells every 24 hours following Dox administration, and then analyzed both precursor and total RNA levels by quantitative RT-PCR. For all three RNAs tested (*GAPDH* mRNA, U2 snRNA, and U4 snRNA), the ratio of unprocessed RNA to total RNA gradually increased as Ssu72 was depleted (Figs. 4B–4D). The ratios 4 days upon Dox addition were 2- to 4-fold higher than the ratios before Dox addition. Importantly, the levels of total RNAs were not significantly influenced by Dox treatment (Fig. S1). These data suggest that vertebrate Ssu72, like its yeast ortholog, is required for efficient 3′-end formation of at least some snRNAs and poly(A)^+^ mRNA. This is the first demonstration that vertebrate Ssu72 participates in 3′-end formation of Pol II transcripts *in vivo*.

### Ssu72 suppresses 3′-end formation of replication-dependent histone mRNAs in DT40 cells

**Figure 5:**
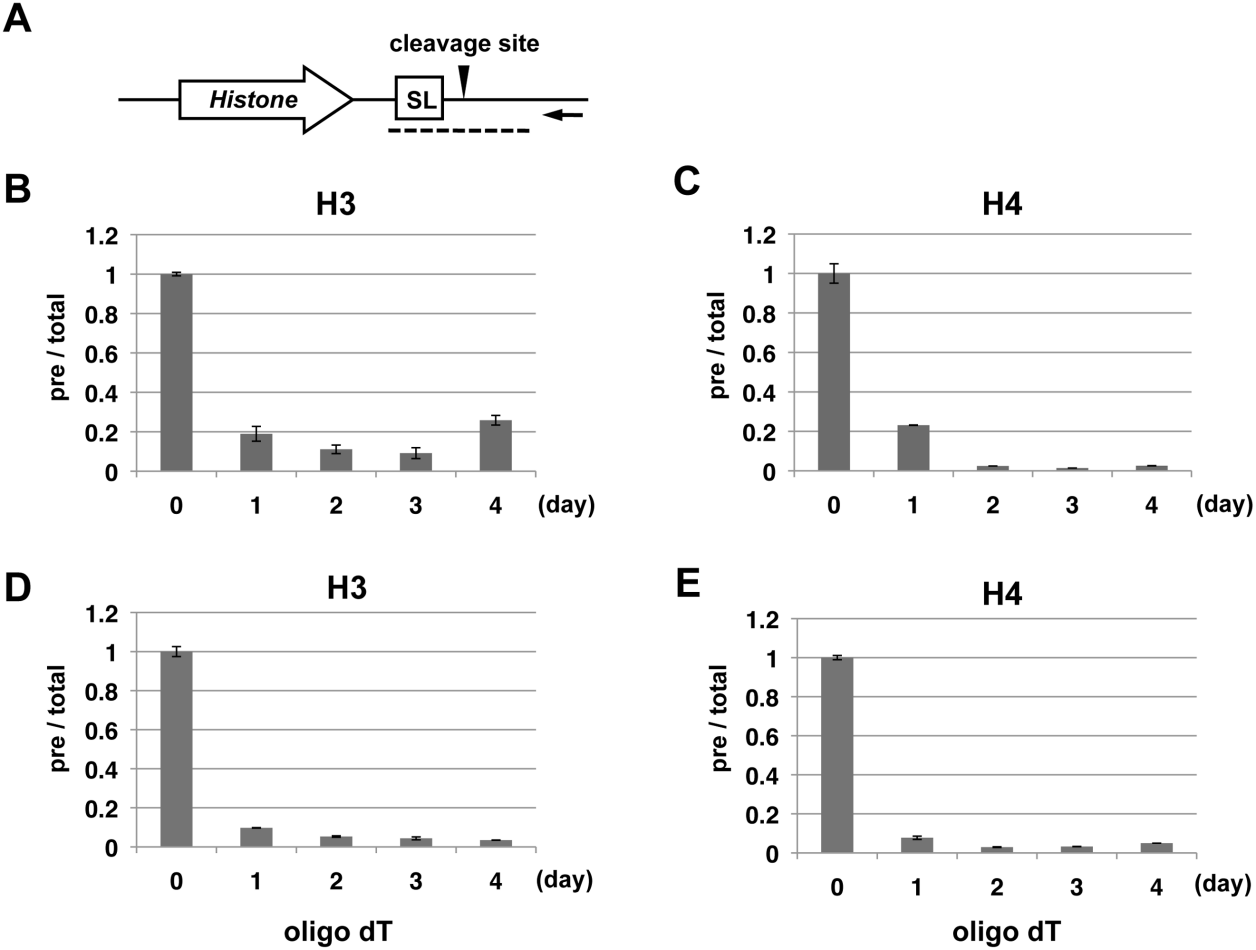
Ssu72 inhibits 3′-end formation of replication-dependent histone mRNAs. (A) Diagram of the histone gene, shown as an open arrow, with the cleavage site depicted an arrowhead and the stem-loop sequence (SL) indicated by the open box. The black arrow represents the gene-specific primer used for reverse transcription of precursor transcripts. Dotted lines indicate the RT-qPCR amplicons used to quantitate the precursor transcripts. (B-E) RT-qPCR analysis of the relative expression levels of precursor (pre) and total transcripts of histone H3 (B, D) or H4 (C, E) in DT40 P3 (−/−) cells treated with Dox for the indicated number of days. Gene-specific primers (B, C) or an oligo-dT primer (D, E) was used for reverse transcription to detect the expression levels of precursor transcripts. Relative expression levels were normalized the corresponding levels on day 0. Error bars represent standard deviations of at least two independent expriments.

The results described above suggest that the functions of Ssu72 in 3′-end formation of snRNAs and mRNAs are evolutionarily conserved from yeast to chicken. The 3′ ends of most mRNAs are formed by cleavage and polyadenylation. However, in metazoans but not yeast, the 3′ ends of replication-dependent histone mRNAs are generated by a distinct process [33]. Primary histone mRNAs are cleaved just downstream of a conserved 3′ stem-loop sequence, but no poly(A) tail is added at the 3′ end of the upstream cleavage product (Fig. 5A). Recent studies showed that regulation of CTD phosphorylation participates in 3′-end processing of replication-dependent histone pre-mRNA [16]. Therefore, we examined whether Ssu72 is involved in 3′-end formation of histone mRNAs (Figs. 5B–5E). Using the assay described above, we used quantitative RT-PCR to determine the ratios of precursor mRNAs to total mRNAs of chicken histone H3 and H4 genes following Ssu72 depletion. Surprisingly, in contrast to the cases of snRNAs and polyadenylated mRNAs, the ratios of precursor RNAs to total RNAs for both H3 and H4 mRNAs dramatically decreased upon depletion of Ssu72 (Figs. 5B and 5C). Notably, the relative precursor levels dropped rapidly, mirroring the reduction in Ssu72 protein level (Fig. 3). Even 1 day after Dox addition, the precursor ratio had decreased to ∼20% of the ratio in untreated cells. Importantly, the levels of total RNAs (precursor + mature) were not dramatically influenced by Dox treatment (Fig. S2). Thus, Ssu72 depletion results in a significant increase in the efficiency of 3′-end formation of histone mRNAs.

In vertebrates, some replication-dependent histone genes contain not only a stem-loop sequence but also a typical polyadenylation signal, generally located downstream of the stem-loop sequence. When histone-specific processing is inhibited or regulated under certain cellular conditions, a portion of transcripts from these histone genes are processed by cleavage and polyadenylation at the downstream polyadenylation signal [16], [34]. To further investigate the possible involvement of Ssu72 in 3′-end formation of histone mRNAs, we examined the effect of Ssu72 depletion on the level of polyadenylated histone H3 and H4 mRNAs, which can be detected using oligo dT-primed first-strand cDNAs. Although polyadenylated histone mRNAs were detectable in the absence of Dox (Figs. 5D and 5E, day 0), their levels quickly decreased after Dox addition (days 1–4), with kinetics similar to those of the relative precursor levels. These results imply that Ssu72 intrinsically suppresses 3′-end formation of stem-loop–dependent histone mRNAs in vertebrates.

### Ssu72 depletion causes hyperphosphorylation of the CTD on Pol II-transcribed genes

**Figure 6:**
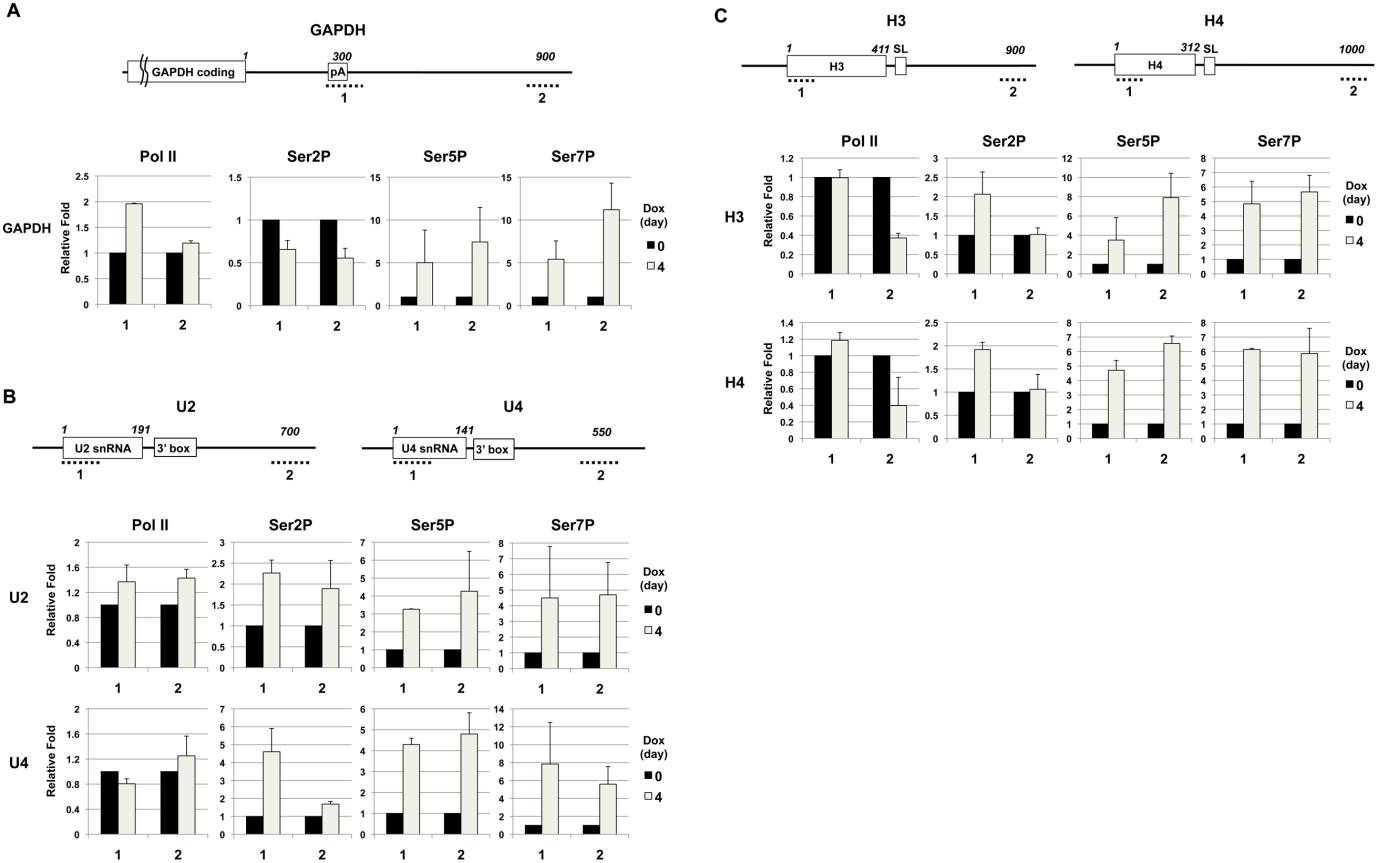
Ssu72 depletion causes hyperphosphorylation of the CTD on Pol II–transcribed genes. (A–C) ChIP analysis of the downstream region of the *GAPDH* gene (A), U2 and U4 snRNA genes (B), and histone H3 and H4 genes (C). (Upper) Diagrams of these genes with the coding regions, polyadenylation signals (pA), 3′ box signals, and stem-loop sequences (SL) shown as open boxes. The numbers above these genes represent distance (in nucleotides) from the end of the coding region (for *GAPDH*) or the transcription start site (for snRNA and histone genes). Dotted lines indicate the ChIP amplicons. (Lower) ChIP analysis of region 1 or 2 on the indicated genes, using antibodies against Pol II (N20), Ser2P (3E10), Ser5P (3E8), or Ser7P (4E12), in DT40 P3 (−/−) cells treated with Dox for 4 days (grey bars) or untreated (black bars). The y axe represents the fold change relative to the corresponding levels on day 0. The values for Ser2P, Ser5P or Ser7P signals were normalized by the total Pol II signal. Error bars represent standard deviations of two independent experiments.

The results described above suggest that vertebrate Ssu72 is required for efficient 3′-end formation of at least some snRNAs and poly(A)^+^ mRNAs, but inhibits 3′-end formation of replication-dependent histone mRNAs. Numerous studies have shown that regulation of CTD phosphorylation plays an important role in 3′-end formation of most Pol II transcripts [5]. Although Ssu72 depletion exhibited only a modest effect on the phosphorylation levels of total Pol II in whole-cell extracts of DT40 cells, we hypothesized that the effects of Ssu72 inactivation on 3′-end formation of Pol II-transcribed genes were caused by aberrant CTD phosphorylation on the affected genes. Therefore, we next examined the effect of Ssu72 depletion on the phosphorylation patterns of Pol II on the affected genes. In chromatin immunoprecipitation (ChIP) analyses using well-characterized phosphorylated CTD-specific antibodies (Fig. 6), we compared the distributions of total and phosphorylated Pol II at two distinct regions on the affected genes before and after Dox treatment of P3 (−/−) cells (Figs. 6A–6C). To evaluate the phosphorylation levels of Ser2, Ser5, and Ser7 on the affected genes, we calculated the ratios of the ChIP signals obtained using phosphorylated CTD-specific antibodies to the signals obtained using antibodies against the N-terminal portion of Rpb1 (total Pol II). We first examined the Pol II distribution in the chicken *GAPDH* gene (Fig. 6A). Four days after Dox addition, when the 3′-end formation of three classes of Pol II transcripts was significantly affected (Figs. 4 and 5), the total Pol II level was elevated by 2-fold near the poly(A) signal sequence (region 1) (Fig. 6A, Pol II). Remarkably, phosphorylation of Ser5 increased about 5-fold in region 1 and region 2 (Fig. 6A, Ser5P); likewise, Ser7P levels increased 5-fold in region 1 and 11-fold in region 2 (Fig. 6A, Ser7P). By contrast, Ser2 phosphorylation decreased slightly in both regions (Fig. 6A, Ser2P).

We next examined the distribution of Pol II on the chicken U2 and U4 snRNA genes (Fig. 6B). Upon Ssu72 depletion, the phosphorylation levels of all serine residues were significantly elevated in 5′ regions (region 1). In the region downstream of the 3′-end processing site (region 2), phosphorylation of Ser5 and Ser7 was elevated to a greater extent (4- to 5-fold) than phosphorylation of Ser2 (less than 2-fold) (Fig. 6B, Ser2P, Ser5P, and Ser7P). We then analyzed the distribution of Pol II on the replication-dependent histone H3 and H4 genes in the 5′ portion of the coding region (region 1) and 1 kb downstream of the first codon (region 2) (Fig. 6C). Ssu72 depletion caused a significant increase in phosphorylation of all serine residues in the 5′ coding regions of both histone genes (Fig. 6C, region 1). Notably, phosphorylation of Ser5 and Ser7 was elevated more than Ser2P levels. On the other hand, in the downstream regions, phosphorylation was markedly increased at Ser5 and Ser7, but not at Ser2 (Fig. 6C, region 2). Furthermore, Ssu72 depletion also led to a significant decrease in Pol II levels in the downstream regions, relative to the 5′ regions (Fig. 6C, Pol II, region 2), indicating that Ssu72 inactivation leads to increased efficiency of transcription termination at the 3′ ends of these histone genes.

Recent studies by Xiang *et al*. and our results shown below demonstrated that human Ssu72, like its yeast counterpart, can dephosphorylate Ser5P and Ser7P *in vitro* [27]. Thus, the ChIP results suggest that Ssu72 maintains the appropriate phosphorylation status of the CTD on different classes of Pol II-transcribed genes by directly dephosphorylating both Ser5P and Ser7P during transcription. To our knowledge, this is the first study to demonstrate that vertebrate Ssu72 plays a role in regulating CTD phosphorylation *in vivo*.

### Vertebrate Ssu72 can dephosphorylate Ser5P and Ser7P, but not Thr4P, *in vitro*

**Figure 7:**
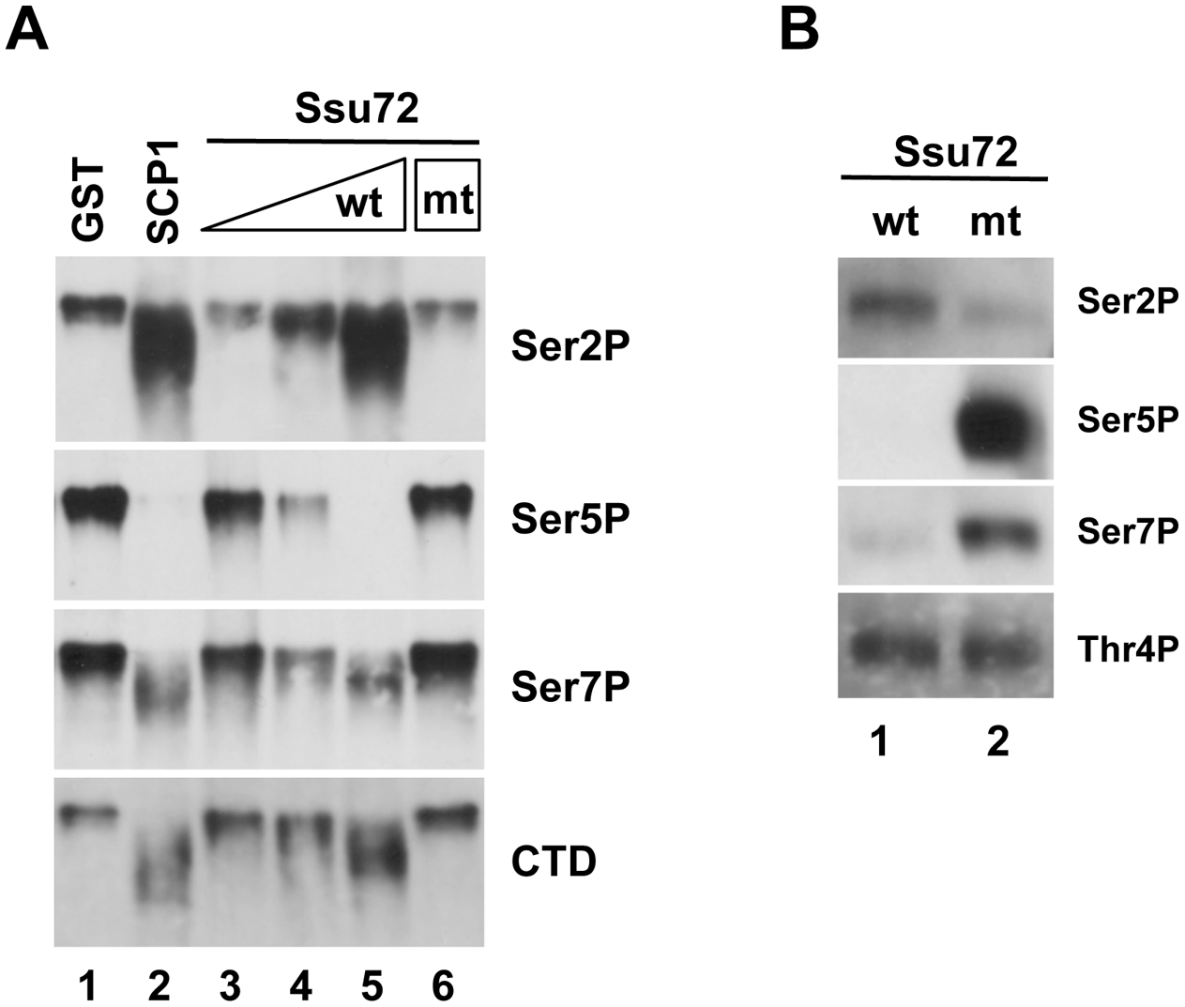
Ssu72 dephosphorylates Ser5P and Ser7P *in vitro*. (A) Fifty ng phosphorylated GST-CTD was incubated with 3.4 µg GST (lane 1), 0.2 µg SCP1 (lane 2), 0.2 µg, 1 µg, or 5 µg wild-type human Ssu72 (lanes 3–5), or 5 µg mutant (C12S) Ssu72 (lane 6), followed by Immunoblotting using the phospho CTD–specific antibodies 3E10 (Ser2P), 3E8 (Ser5P), and 4E12 (Ser7P) and an antibody against the CTD (8WG16). (B) Purified human Pol II was incubated with wild-type (lane 1) or mutant (C12S) Ssu72 (lane 2) and analyzed by Western blotting using phospho CTD–specific antibodies 3E10 (Ser2P), 3E8 (Ser5P), 4E12 (Ser7P), and 6D7 (Thr4P).

The elevation of Ser2P both in the 5′ portion of snRNAs and the coding region of histone genes upon Ssu72 inactivation (Figs. 6B and 6C) raised the possibility that Ssu72 can also directly dephosphorylate Ser2P. To investigate this issue, we tested whether purified recombinant Ssu72 could dephosphorylate Ser2P in an *in vitro* dephosphorylation assay using GST-fused phosphorylated CTD as a substrate (Fig. 7) [35]. The CTD dephosphorylation was monitored by Western blot using phosphorylation-dependent CTD antibodies used in Figure 3. In this experiment, we used recombinant human Ssu72 protein because it is almost identical to chicken Ssu72 (99% identity), and the two proteins have the same specific activity. We also used, as a positive control, recombinant human small CTD phosphatase 1 (SCP1), which belongs to a family of the FCP1-like enzyme and preferentially dephosphorylates Ser5P [36], [37]. As expected, SCP1 has strong phosphatase activity towards Ser5P and, to a lesser extent, towards Ser7P, whereas no activity was detected towards Ser2P (Figure 7A, lane 2). Increasing amounts of bacterially-expressed and purified human Ssu72 efficiently dephosphorylated both Ser5P and Ser7P, but did not affect Ser2P; instead, the phosphorylation level of Ser2 increased (Fig. 7A). The apparent increase in Ser2P may have been caused by the increased affinity of our Ser2P-specific antibody (3E10) for CTDs dephosphorylated at both Ser5P and Ser7P, relative to its affinity for CTDs phosphorylated at either residue. This property of the 3E10 antibody may account for some changes of Ser2P ChIP signals obtained in the above experiments (Figure 6).

We next examined the activity and specificity of human Ssu72 for CTD dephosphorylation using the Pol II core complex purified from HeLa cells, which is a more biologically relevant substrate. Wild-type Ssu72, but not a catalytically dead mutant (C12A), efficiently dephosphorylated Ser5P and Ser7P. However, wild-type Ssu72 did not exhibit any phosphatase activity toward Ser2P (Fig. 7B). These results clearly demonstrate that human Ssu72 possesses Ser5P/Ser7P but not Ser2P phosphatase activity *in vitro*. Therefore, the elevation of Ser2P in the 5′ regions of snRNA and histone genes may be caused by abnormally high levels of Ser5 and Ser7 phosphorylation, which may indirectly affect the Ser2P levels, possibly by inhibiting a Ser2P phosphatase or activating a Ser2 kinase only in the promoter regions of specific genes.

A recent study using a DT40 cell line bearing a conditional knockout of Rpb1 revealed that Thr4P is required for efficient histone mRNA 3′-end processing [16]. This observation raised the possibility that Ssu72 plays some inhibitory roles in histone mRNA 3′-end processing by directly dephosphorylating Thr4P. Therefore, we investigated whether human Ssu72 has Thr4P phosphatase activity, using the phosphatase assays described above and an antibody specific for Thr4P (6D7) [38]. After incubation with either wild-type Ssu72 or the catalytically inactive mutant, Thr4P levels of purified Pol II were not significantly altered (Fig. 7B, Thr4P). Thus, human Ssu72 has no phosphatase activity toward Thr4P *in vitro*.

## Discussion

In this study, we established Ssu72 conditional-knockout chicken DT40 cell lines and used them to investigate the functions of Ssu72 in gene expression at the cellular level. Our results demonstrated that chicken Ssu72, similar to its yeast ortholog, is essential for cell viability and efficient 3′-end formation of at least some snRNAs, as well as polyadenylated mRNAs. Unexpectedly, however, inactivation of Ssu72 caused a rapid and marked decrease in the levels of unprocessed replication-dependent histone mRNAs, indicating that chicken Ssu72 normally suppresses the stem-loop–type 3′-end formation of histone mRNA. Furthermore, Ssu72 inactivation led to a marked increase in both Ser5 and Ser7 phosphorylation on all genes in which 3′-end formation was affected. This is the first demonstration that Ssu72 functions as a Ser5P/Ser7P phosphatase in vertebrate cells. Together, our findings suggest that vertebrate Ssu72 is involved in various ways in 3′-end formation of Pol II transcripts, and that its function is mediated by dephosphorylation of CTD at both Ser5 and Ser7.

### Chicken Ssu72 is essential for cell proliferation

Our results demonstrated that chicken Ssu72 is indispensable for cell proliferation (Fig. 2). Although Ssu72 depletion affected 3′-end formation of particular snRNAs and mRNAs, it did not significantly influence the steady-state levels of these RNAs (Figs. S1 and S2). At present, we do not know which aspects of Ssu72 functions are required for cell viability. A recent report showed that replacement of the Ser7 residue with phospho-mimetic glutamate in all CTD heptapeptide repeats is lethal, both in budding yeast [24] and in mammals [32], suggesting that persistent elevation of the Ser7P level dramatically decreases cell viability. As shown in this study, Ssu72 depletion results in dramatic elevation of Ser7P levels on several Pol II genes, even though depletion exerts only a modest effect on overall levels of Ser7P (Figs. 3). We assume that Ssu72 depletion significantly suppresses the expression of particular genes, possibly including genes essential for cell proliferation, by sustaining high Ser7P levels on those genes. To identify such specifically affected genes, global gene expression analysis will be required.

Alternatively, apart from its possible function in gene expression, the essential nature of Ssu72 in vertebrates might be explained by the recent findings of Kim et al [39]. Their results indicated that human Ssu72 might regulate the resolution of sister chromatid arm cohesion through direct interactions with the cohesion subunits Rad21 and SA2. Because we did not investigate whether Ssu72 depletion causes abnormalities in sister chromatid cohesion in DT40 cells, our results cannot elucidate whether the lethality of Ssu72-deficient cells can be attributed to such defects.

### Chicken Ssu72 stimulates 3′-end formation of snRNAs and polyadenylated mRNAs

In this study, we demonstrated that chicken Ssu72 inactivation resulted in inefficient 3′-end formation of both snRNAs and polyadenylated mRNAs (Fig. 4), concomitant with elevation of Ser5P and Ser7P levels in the 3′ regions of these genes (Fig. 6A and 6B). Our results are in accordance with a study of cultured human cells by Egloff et al. [15] showing that substituting either the Ser5 or Ser7 residue with phospho-mimetic glutamate (Ser5E or Ser7E) in all CTD heptapeptide repeats led to defects in 3′-end formation of both snRNAs and polyadenylated mRNAs [15]. Why does elevation of either Ser5P or Ser7P levels cause the defects of 3′-end formation of these types of Pol II-transcribed genes? For polyadenylated mRNAs, our ChIP results demonstrated that Ssu72 inactivation also resulted in reduction of Ser2P levels at the 3′-end processing site and downstream region of the *GAPDH* gene (Fig. 6A). This observation is also consistent with the study by Egloff et al., who showed that either the Ser5E or Ser7E mutation resulted in a dramatic decrease in Ser2P levels [15]. These results indicate that aberrant elevation of either Ser5P or Ser7P may somehow decrease the level of Ser2P, which is required for the efficient recruitment of 3′-end processing machinery to the 3′ regions of polyadenylated mRNAs.

In metazoans, 3′-end processing of snRNA requires the Integrator complex, which recognizes the 3′ box sequence just downstream of the processing site and binds CTD phosphorylated at both Ser2 and Ser7 (Fig. 4A) [40], [41]. A recent study suggested that the Integrator complex is recruited to elongating Pol II via an interaction with a putative Ser5P-phosphatase, RPAP2, which recognizes the Ser7P mark [14]. Therefore, the elevation in Ser7P resulting from Ssu72 inactivation could potentially promote the recruitment of RPAP2 and the Integrator complex to Pol II–transcribed snRNA genes. However, simultaneous elevation of Ser7P and Ser5P levels caused by Ssu72 depletion might interfere with the interaction between the CTD and Integrator complex, as implied in a previous study [40]. We hypothesize that dephosphorylation of both Ser5P and Ser7P, are carried out either Ssu72 and RPAP2 or by Ssu72 alone, is required for the efficient 3′-end formation of vertebrate snRNAs.

### Chicken Ssu72 suppresses 3′-end formation of replication-dependent histone mRNAs

To our surprise, we observed that Ssu72 depletion dramatically decreased levels of the unprocessed form of replication-dependent histone pre-mRNAs in DT40 cells (Fig. 5). We interpret these results as indicating that Ssu72 normally suppresses 3′-end formation of histone mRNA; consequently, Ssu72 inactivation leads to de-repression. Consistent with this view, our ChIP experiments revealed reductions in Pol II occupancies downstream of the processing site of both H3 and H4 genes (Fig. 6C), indicating that the efficiency of processing and/or transcription termination was higher in Ssu72-depleted cells.

Interestingly, a recent genome-wide ChIP-seq study in mammalian cells demonstrated that profiles of Pol II occupancy downstream of 3′-end processing site of protein-coding genes markedly differed between core histone genes and polyadenylated genes [42]. The Pol II occupancy downstream of 3′-end processing site of core histone genes exhibited sharp drop whereas those of polyadenylated genes persisted throughout much longer region, suggesting that difference in 3′-end processing mechanism of these RNAs influence the transcription termination process [42]. We assume that Ssu72 may be a key factor determining which types of 3′-end formation process is selected during transcription by Pol II.

How does Ssu72 specifically suppress histone mRNA 3′-end formation while playing a positive role in 3′-end formation in other types of Pol II–transcribed RNAs? The 3′-ends of most protein-coding mRNAs are formed by a two-step reaction consisting of an endonucleolytic cleavage and a tightly-coupled polyadenylation. However, in metazoan but not yeast, the 3′-ends of replication-dependent histone mRNAs are processed by different mechanism, in which precursor RNAs are endonucleolytically cleaved at a site between a conserved stem-loop sequence and a histone gene-specific downstream element recognized by U7 snRNP but are not followed by polyadenylation. Despite distinct complexes participate in each type of processing reaction, histone mRNA 3′-end formation shares many factors with the canonical polyadenylation reaction, including the endonuclease CPSF73 [33]. Another common factor is symplekin, the metazoan ortholog of budding yeast Pta1, which functions as a scaffold protein for both types of 3′-end processing [33]. Both symplekin and Pta1 strongly bind Ssu72, via an interaction with its N-terminal domain (NTD). Multiple authors have suggested that the NTD exerts an inhibitory effect on 3′-end processing of poly(A)-containing mRNAs, both in budding yeast and human, and that Ssu72 promotes 3′-end processing of polyadenylated mRNAs by counteracting the negative effects of symplekin/Pta1 [28], [43]. Inversely, we speculate that vertebrate Ssu72 inhibits histone 3′-end processing by counteracting a positive effect of symplekin.

In recent study using the DT40 conditional-knockout system, replacement of Thr4 with Val in all repeats of the CTD caused a defect in efficient 3′-end processing, but not in transcription of replication-dependent histone mRNAs [16]. Moreover, inhibition of CDK9 activity in chicken or human cells compromised the 3′-end processing of histone mRNAs [34] and reduced the level of Thr4P [16], suggesting that Thr4P is involved in 3′-end processing of histone mRNA. Based on our observation (Fig. 7) that Ssu72 did not exhibit any phosphatase activity toward Thr4P *in vitro*, it is less likely that Ssu72 inhibits histone mRNA processing by directly dephosphorylating Thr4P. Instead, Thr4P may inhibit dephosphorylation of Ser5P and/or Ser7P by Ssu72 and thereby interfere with the suppressive activity of Ssu72 in histone mRNA processing. This possibility is consistent with a recent finding that the *in vitro* phosphatase activity of *Drosophila* Ssu72 toward Ser5P is reduced 4-fold by Thr4 phosphorylation within the same repeat [31]. Further studies will be required to decipher the underlying mechanism.

### Chicken Ssu72 functions as a Ser5P and Ser7P phosphatase on Pol II–transcribed genes *in vivo*

Recent *in vitro* studies by Xiang et al. demonstrated that human Ssu72 exhibits CTD phosphatase activity toward Ser5P and Ser7P [27], [28]. Their results showed that whereas Ser7P phosphatase activity of Ssu72 was much lower (∼4000-fold) than its Ser5P phosphatase activity when CTD phosphopeptide was used as a substrate, Ssu72 exhibited comparable phosphatase activity toward Ser5P and Ser7P on full-length mammalian CTD. This observation suggests that multiple heptad repeats or non-consensus heptad repeats somehow help Ssu72 to recognize its substrate or catalyze dephosphorylation of Ser7P. Consistent with these findings, our results in this study demonstrated that chicken Ssu72 inactivation led to a comparable increase in both Ser5 and Ser7 phosphorylation on all affected genes (Fig. 6). Furthermore, our ChIP results showed that the fold increase in Ser7P levels after Ssu72 depletion was much higher than the increase in Ser5P levels in the downstream regions of *GAPDH* (Figs. 6A), indicating that CTD phosphatase activity of Ssu72 toward Ser7P may be specifically activated in the 3′ regions of at least some Pol II–transcribed genes. A recent study showed that the N-terminal region of the metazoan mRNA 3′-end processing factor symplekin stimulates, to similar extents, both the Ser5P and Ser7P phosphatase activity of Ssu72 *in vitro* [27]. Therefore, preferrential activation of Ssu72 phosphatase activity towards Ser7P may be caused by association of novel factors other than symplekin, e.g., transcription termination factors. Alternatively, a conformational change of the CTD, such as proline isomerization, in the 3′-end region may facilitate dephosphorylation of Ser7P by Ssu72. In any case, our study provides the first demonstration that Ssu72 functions as a Ser5P/Ser7P phosphatase in vertebrate cells.

Although Ssu72 depletion in budding yeast resulted in a significant increase in the Ser5P level of total Pol II *in vivo* [23], both Ser5P and Ser7P levels of total Pol II in DT40 cells were slightly increased by Ssu72 depletion, indicating that other Ser5 and Ser7 phosphatases may exist in vertebrate cells. Indeed, in mammals, the small CTD phosphatase SCP1 has been reported to exhibit Ser5P-specific phosphatase activity *in vitro* [36], [37]. FCP1 (TFIIF-associating CTD phosphatase), considered to be a Ser2P-specific phosphatase [44], [45], can also efficiently dephosphorylate Ser5P *in vitro* [27], [46]. Furthermore, SCP1 (Fig. 7A), and FCP1 [27] exert potent CTD phosphatase activity towards Ser7P *in vitro*. These observations imply that Ser5P and Ser7P levels of total cellular Pol II in vertebrate cells are either primarily regulated by CTD phosphatases other than Ssu72 or redundantly regulated by several CTD phosphatases including Ssu72. Nevertheless, our results suggest that Ssu72 functions as a Ser5/7P phosphatase during the transcription of a subset of Pol II genes. We speculate that after Ssu72 ceases to act or dissociates from Pol II, other CTD phosphatases such as FCP1 or SCP1 remove most of the remaining phosphate groups from Ser2/5/7P before the next round of transcription.

### Ssu72-knockout DT40 cells provide a valuable tool for studying gene looping in vertebrate cells

As a component of the budding yeast pre-mRNA 3′-end processing complex, Ssu72 is required for the formation of gene loops [47], which are established by physical interactions between promoters and terminators of transcribed genes [48]. Gene loops have been proposed to function in transcription re-initiation and transcriptional memory [47], [49]. Furthermore, Tan-Wong et al. recently showed that Ssu72-mediated gene-loop formation in budding yeast is restricted the production of divergent non-coding transcripts from bidirectional promoters, indicating that gene loops help to maintain the appropriate directionality of transcription [50]. Furthermore, they demonstrated in a human cell line that a polyadenylation-signal mutation of an integrated artificial β-globin gene resulted in suppression of both gene-loop formation and induction of divergent transcription, indicating that gene looping and its role in maintaining transcription directionality are conserved phenomena [50]. Although gene looping has also been detected in HIV-1 provirus [51] and some mammalian endogenous genes such as *CD68* [52] and *BRCA1* [53], the factors involved in gene-loop formation in vertebrate cells remain unknown. The Ssu72-knockout DT40 cells we established may provide a valuable tool for studying gene looping in vertebrate cells.

## Experimental Procedures

### Cell culture and transfection

Chicken DT40 cell lines were provided by Dr. K. Yamamoto (Department of Molecular Pathology, Cancer Research Institute, Kanazawa University, Kanazawa, Japan) [54]. DT40 cells were cultured in RPMI 1640 medium (Nissui) supplemented with 10% fetal bovine serum (Cell Culture Bioscience), 1% chicken serum (Gibco), 50 mM 2-mercaptoethanol (Sigma), penicillin, streptomycin, and 2 mM L-glutamine at 39°C in a humidified 5% CO_2_ incubator. Transfections were carried out by electroporation using a GENE Pulser II (Bio-Rad) at 25 mF and 550 V. Drug-resistant clones were selected with medium containing 1.5 mg/ml G418, 25 µg/ml blasticidin S, 0.5 µg/ml puromycin, or 2.5 mg/ml hygromycin.

### Plasmid constructs

Two genomic DNA fragments, a 2 kb fragment upstream of the first exon of the Ssu72 gene (ENSGALG00000001489) and a 3.9 kb fragment downstream of the second exon, were amplified from DT40 genomic DNA by long-range PCR. These fragments were cloned into the flanking regions of the drug-resistance cassettes (puromycin or blasticidin S) [55], which replaced the genomic region spanning the first and second exons (Fig. 1). The full-length chicken Ssu72 cDNA was amplified by RT-PCR from DT40 total RNA, and then cloned into the doxycycline-inducible vector supplied in the Tet-Off Advanced Inducible Gene Expression System (Clontech). Plasmids expressing GST-fused human SCP1 were prepared essentially as described [35]. For construction of the plasmid expressing His-tagged human Ssu72 in *E. coli*, the full-length human Ssu72 cDNA was amplified by RT-PCR from HeLa cell total RNA using primer set: hSsu72ATG-KP and hSsu72TGA-SL, and then cloned into a Kpn I/Sal I-digested pCold-II vector (TaKaRa). The plasmid expressing the mutant human Ssu72 (C12S) was made by site-directed mutagenesis using the KOD Plus Mutagenesis Kit (TOYOBO). Sequences of all cDNA inserts derived from PCR amplification were verified by DNA sequencing.

### Generation of mutant DT40 cells

Wild-type DT40 cells were transfected with the pTet-off vector (Clontech), and drug-resistant clones were selected by culturing in G418-containing medium. One of the drug-resistant clones constitutively expressing tetR-VP16 protein was selected and used to establish stable clones expressing chicken Ssu72. In the resultant clones, Ssu72 expression is tightly repressed by addition of tetracycline. One of those clones was then used to generate the conditional Ssu72-deficient DT40 cell lines by sequentially introducing two types of linearized targeting constructs. At each step of gene disruption, genomic DNA was isolated from drug-resistant clones and subjected to Southern blotting and genomic PCR to confirm gene replacement by homologous recombination.

### Southern blotting

Genomic DNA isolated from drug-resistant clones was digested with *Eco*RI and *Xba*I, and the resultant fragments were electrophoresed in 1.0% agarose gels and transferred to nylon membranes. Blots were hybridized with a ^32^P-labeled 5′ DNA probe (see Fig. 1A) and analyzed by autoradiography.

### Growth curves and cell-cycle distribution analysis

Cells were seeded in triplicate in 12-well plates (5×10^3^ cells/well) and cultured under normal conditions for 8 days, during which they were diluted at 2-day intervals. Every day, concentrations of live cells were determined by Giemsa staining and counting on a hemocytometer. To examine cell-cycle distribution, 1–5×10^6^ cells were fixed in 70% ethanol, treated with RNase A, and stained with propidium iodide. DNA contents were measured on a FACSCalibur instrument, and cell-cycle profiles were analyzed using the CellQuest software (BD Biosciences).

### Immunoblotting and antibodies

Immunoblotting was performed as described previously [35]. The following antibodies were used: phosphorylation-specific anti-CTD [3E8 (pSer2), 3E10 (pSer5), 4E12 (pSer7), and 6D7 (pThr4)] (Ascenion); anti-Rpb1 (ARNA-3, PROGEN); anti-β-actin (Sigma); and anti-DNA polymerase delta and anti-exportin-1 (Transduction). Affinity-purified anti-Ssu72 antibodies were prepared by our laboratory.

### RT-PCR

Total RNA was isolated from wild-type and mutant DT40 cells using TRIzol (Invitrogen). First-strand cDNA was synthesized from total RNA using Superscript III reverse transcriptase (Invitrogen) with random hexamer primers. The indicated cDNAs were amplified by PCR using specific primers, and then analyzed by agarose gel electrophoresis followed by staining with SYBR Green I (Invitrogen). For RT-qPCR, total RNA (500 ng) purified from DT40 cells using the Nucleo Spin RNA II kit (TaKaRa) was subjected to reverse transcription (RT) using the PrimeScrip RT Master Mix (TaKaRa). Synthesized first-strand cDNA was quantitated using SYBR Premix Ex Taq II (TaKaRa) on an Mx3000P Real-Time PCR System (Stratagene).

### Chromatin immunoprecipitation analysis (ChIP)

Cells (1×10^7^) cultured in medium were cross-linked by addition of formaldehyde (final concentration, 1%); after incubation for 10 minutes at room temperature, the reaction was quenched with 125 mM glycine. Cells were washed twice with 10 ml of ice-cold PBS, resuspended in 250 µl of SDS Lysis buffer (50 mM Tris-HCl [pH 8.1], 10 mM EDTA, and 1% SDS) containing protease inhibitor cocktail, and incubated for 10 minutes on ice. Cell lysates were sonicated using a Bioruptor UCD-200 (Cosmobio) to generate DNA fragments of 200–500 bp. Samples were centrifuged for 20,000× *g* for 10 minutes, and the resultant supernatant was diluted 10-fold with Dilution buffer (16.7 mM Tris-HCl [pH 8.1], 167 mM NaCl, 1.2 mM EDTA, 1.1% Triton X-100) containing protease inhibitor cocktail; 500 µl of the diluted sample was mixed with antibodies and rotated overnight at 4°C. The following day, 90 µg of Dynabeads Protein G (Invitrogen), pre-coated with salmon sperm DNA, was added to the mixture. The sample was then incubated for 1.5 hours at 4°C, and then washed twice with 1 ml of Low Salt buffer (20 mM Tris-HCl [pH 8.1], 150 mM NaCl, 2 mM EDTA, 0.1% SDS, and 1% Triton X-100), twice with High Salt buffer (20 mM Tris-HCl [pH 8.1], 500 mM NaCl, 2 mM EDTA, 0.1% SDS, and 1% Triton X-100), and once with LiCl buffer (10 mM Tris-HCl [pH 8.1], 250 mM LiCl, 1 mM EDTA, 1% sodium deoxycholate, and 1% Nonidet P-40). After the final wash, the beads were agitated with 200 µl of Elution buffer (100 mM NaHCO_3_ and 1% SDS) at room temperature for 30 minutes. To reverse cross-links, 8 µl of 5 M NaCl was added, and the samples were incubated at 65°C overnight. The following day, the samples were incubated with 10 µg of RNase A at 37°C for 1 hour, and then with 10 µg of Proteinase K at 45°C for 2 hours. DNA fragments were purified using Wizard SV Gel and the PCR Clean-Up System (Promega), and subjected to RT-PCR using SYBR Premix Ex Taq II (TaKaRa) on an Mx3000P Real-time PCR system.

### Protein expression and purification

The cold-induced expression of the His-tagged human Ssu72 in *E. coli* was performed according to the instruction of pCold-II vector (TaKaRa). The purification of His-tagged recombinant protein was performed with Ni-NTA agarose beads (QIAGEN) according to the instruction manual. All GST fused recombinant proteins were expressed in *E. coli* and purified as described previously [56]. Purification and separation of the phosphorylated Pol II from HeLa cell nuclear-extract pellets were done as described [56]. Protein concentration was determined by the Bradford method using bovine serum albumin (BSA) as a standard.

### *In vitro* CTD dephosphorylation assay

*In vitro* phosphorylation of GST-CTD by HeLa cell nuclear extracts (NE) was performed as described [56]. CTD phosphatase assay were performed essentially as previously described [35]. Briefly, 50 ng phosphorylated GST-CTD or 25ng purified Pol II was incubated with 400 ng His-tagged human wild-type or C12A mutant Ssu72 in 10 ml of CTD phosphatase buffer (50 mM Tris-HCl [pH 7.9], 10 mM MgCl_2_, 5mM DTT, 5% glycerol, 0.025% Tween 20, and 0.1 mM EDTA) at 30°C for 25 min. The reaction was terminated by addition of SDS loading buffer, and the reaction products were analyzed by immunoblotting with phospho-specific CTD antibodies.

### Primers lists used in this study

Primers for constructing expression plasmids

cSsu72NheATG ACGAATTCGCTAGCCATGCCTTCTTCGCCGCTCCGTG
cSsu72BglTAA CTCTAGATCTTAATAAAAGCAGACAGTATGAAGAAAAGTCCT
hSsu72ATG-KP TACCGGTACCATGCCGTCGTCCCCGCTGCGG
hSsu72TGA-SL GACGTCGACTCAGTAGAAGCAGACGGTGTGCAGAAAG

Primers for RT

GAPDH dw_R2 CAGAACACTTGCTGGAGTTG.
TFIIE-alpha RT-R1 TCCTAAAGGAGGGCCTCCGATGG
U2 R3 GCTCAGCGCAACCGAACGCAG
U4B R3 CCGGCTCCCATTCATCCGTTGCTCAG
H3 R3 TCGCTGCCAGCACAGCGATAC
H4 R3 CAATGGGTGGTCTCTTCAAG

Primers for real-time PCR (RT, total)

GAPDH ex2-3 F TATCTTCCAGGAGCGTGACC
GAPDH ex2-3 R TCTCCATGGTGGTGAAGACA
TFIIE-alpha 2F TCACAGAATGTACAGCGGAAA
TFIIE-alpha GATCCCGATGAAATCAAGGA
U2 F1 ATCGCTTCTCGGCCTTTTGGC
U2 RT-R1 GTCCTCTCATCGAGGACGTATC
U4 F CGGAGAGAAGGGAGGTCAG
U4 R CCTCGGATTAACCTCATTGG
H3 F1 ATGGCGCGTACGAAGCAGACG
H3 R1 CTTGGTGGCCAGCTGCTTG
H4 F1 ATGTCTGGCAGAGGCAAGGG
H4 R1 TGTTGTCGCGCAGCACCTTG

Primers for real-time PCR (RT, precursor)

GAPDH dw1_F TCACCATCTGAAGTGCCTTG
GAPDH dw1_R CAGGGTGGCTGTAAGCATTT
TFIIE-alpha dw1_F CCCTTCGTTGTCTTCCATGT
TFIIE-alpha dw1_R TGGGTTGTTTCCTTGAATCG
U2 F2 CCGGGAGGAATGTGGCGTG
U2 R2 GCGGGACACAGCGCCACCCTC
U4 2F TTGGTGTCCAACAGCAGAAA
U4 2R CCATTCATCCGTTGCTCAG
H3 F2 CTGAGGCTGCTGTGCTTTCAC
H3 R2 GCGATACAGCTCTCCCCAGTG
H4 F2 TCTCTGACGTTAGGCTTGGC
H4 R2 CAGCTCTTTTCCAGGCTAAG

Primers for real-time PCR (ChIP)

GAPDH dw1_F TCACCATCTGAAGTGCCTTG
GAPDH dw1_R CAGGGTGGCTGTAAGCATTT
GAPDH dw2_F AGCTCTGACTGCAGGAGTGG
GAPDH dw2_R CTGGCAGAGGGCTGATACTT
U2 F1 ATCGCTTCTCGGCCTTTTGGC
U2 RT-R1 GTCCTCTCATCGAGGACGTATC
U2 4F CCCCGTCTCCAGCCTGTCCA
U2 4R GGGACCCTGAGCCTGCAGGT
U4 F AGCTTTGCGCAGTGGCAGTATC
U4 R TCGTCATGGCGGGGTATTGG
U4 dw-2F ATCGGCACTGGCTTCACTT
U4 dw-2R CGCGACAAGGAGTTAAGGAG
H3 F1 ATGGCGCGTACGAAGCAGACG
H3 R1 CTTGGTGGCCAGCTGCTTG
H3 dw1_F GGCGCTGATAAGAGAAGTGC
H3 dw1_R TCTGTTCTGAGAGCCACGTC
H4 F1 ATGTCTGGCAGAGGCAAGGG
H4 R1 TGTTGTCGCGCAGCACCTTG
H4 dw1_F GCAAAACCTCCAGTCAACAAA
H4 dw1_R GGACAGCATAGTGGGGAAGA

## Supporting Information

Figure S1 **The levels of total snRNAs and mRNA are not significantly changed by Ssu72 depletion.** (A) The expression levels of chicken U2 snRNA, U4 snRNA, and GAPDH in DT40 P3 (−/−) cells treated with Dox for the indicated days were measured by RT-qPCR analysis. The relative expression levels were normalized to those of 0 day. Error bars indicate standard deviation. (B) The expression levels of chicken U4, U13, and GAPDH in DT40 P3 (−/−) cells treated with Dox for the indicated days were measured by Northern blot analysis probed with the ^32^P-labeled specific cDNA fragments.
(TIF)

Figure S2 **The levels of total histone mRNAs are not dramatically changed by Ssu72 depletion.** The expression levels of chicken H4 and H3 histone mRNAs in DT40 P3 (−/−) cells treated with Dox for the indicated days were measured by RT-qPCR analysis. The relative expression levels were normalized to those of 0 day. Error bars indicate standard deviation.
(TIF)
