# Supplementary figures and images for: Vertebrate Ssu72 Regulates and Coordinates 3′-End Formation of RNAs Transcribed by RNA Polymerase II
Source: PLoS One. 2014 Aug 28;9(8):e106040. doi: 10.1371/journal.pone.0106040 (PMC4148344; doi:10.1371/journal.pone.0106040)

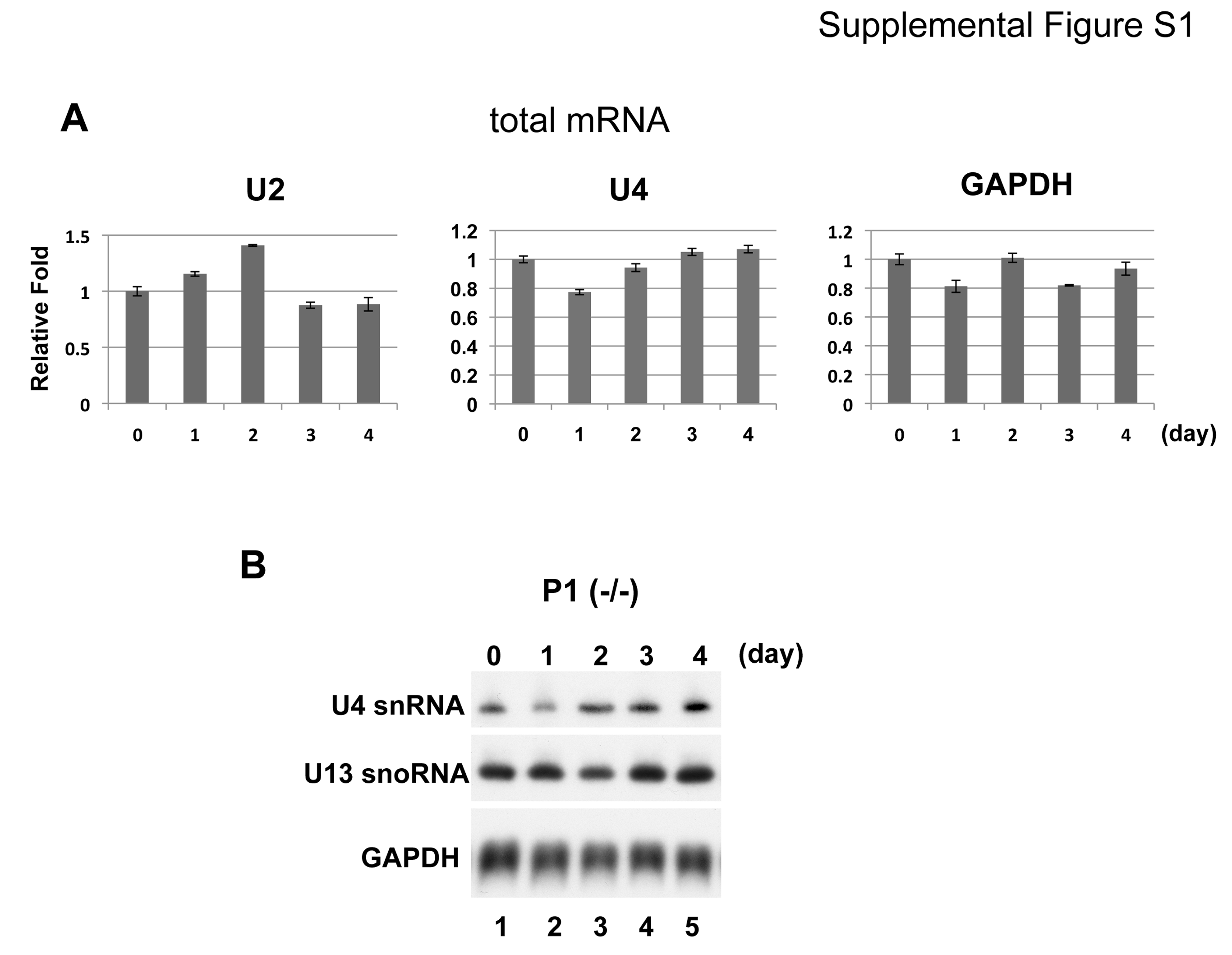

Supplement: Figure S1 — The levels of total snRNAs and mRNA are not significantly changed by Ssu72 depletion. (A) The expression levels of chicken U2 snRNA, U4 snRNA, and GAPDH in DT40 P3 (−/−) cells treated with Dox for the indicated days were measured by RT-qPCR analysis. The relative expression levels were normalized to those of 0 day. Error bars indicate standard deviation. (B) The expression levels of chicken U4, U13, and GAPDH in DT40 P3 (−/−) cells treated with Dox for the indicated days were measured by Northern blot analysis probed with the 32P-labeled specific cDNA fragments. (TIF) [file pone.0106040.s001.tif]

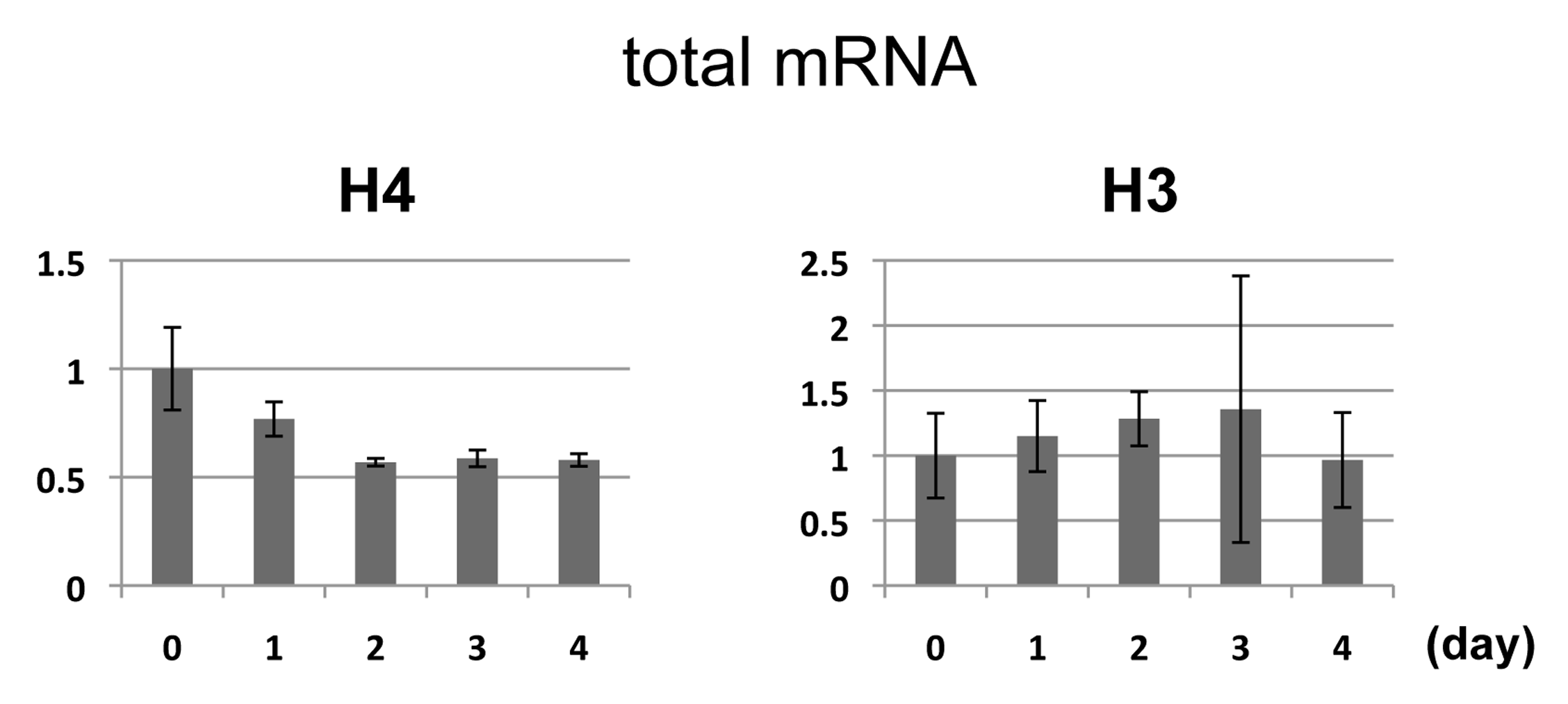

Supplement: Figure S2 — The levels of total histone mRNAs are not dramatically changed by Ssu72 depletion. The expression levels of chicken H4 and H3 histone mRNAs in DT40 P3 (−/−) cells treated with Dox for the indicated days were measured by RT-qPCR analysis. The relative expression levels were normalized to those of 0 day. Error bars indicate standard deviation. (TIF) [file pone.0106040.s002.tif]
